# Differential Evolutionary Selection and Natural Evolvability Observed in ALT Proteins of Human Filarial Parasites

**DOI:** 10.1371/journal.pone.0148611

**Published:** 2016-02-18

**Authors:** Neil C. Devoe, Ian J. Corbett, Linsey Barker, Robert Chang, Polyxeni Gudis, Nathan Mullen, Kailey Perez, Hugo Raposo, John Scholz, Meghan May

**Affiliations:** University of New England College of Osteopathic Medicine, Biddeford, Maine, 04005, United States of America; George Washington University School of Medicine and Health Sciences, UNITED STATES

## Abstract

The abundant larval transcript (ALT-2) protein is present in all members of the *Filarioidea*, and has been reported as a potential candidate antigen for a subunit vaccine against lymphatic filariasis. To assess the potential for vaccine escape or heterologous protection, we examined the evolutionary selection acting on ALT-2. The ratios of nonsynonymous (K(a)) to synonymous (K(s)) mutation frequencies (ω) were calculated for the *alt-2* genes of the lymphatic filariasis agents *Brugia malayi* and *Wuchereria bancrofti* and the agents of river blindness and African eyeworm disease *Onchocerca volvulus* and *Loa loa*. Two distinct Bayesian models of sequence evolution showed that ALT-2 of *W*. *bancrofti* and *L*. *loa* were under significant (*P*<0.05; *P* < 0.001) diversifying selection, while ALT-2 of *B*. *malayi* and *O*. *volvulus* were under neutral to stabilizing selection. Diversifying selection as measured by ω values was notably strongest on the region of ALT-2 encoding the signal peptide of *L*. *loa* and was elevated in the variable acidic domain of *L*. *loa* and *W*. *bancrofti*. Phylogenetic analysis indicated that the ALT-2 consensus sequences formed three clades: the first consisting of *B*. *malayi*, the second consisting of *W*. *bancrofti*, and the third containing both *O*. *volvulus* and *L*. *loa*. ALT-2 selection was therefore not predictable by phylogeny or pathology, as the two species parasitizing the eye were selected differently, as were the two species parasitizing the lymphatic system. The most immunogenic regions of *L*. *loa* and *W*. *bancrofti* ALT-2 sequence as modeled by antigenicity prediction analysis did not correspond with elevated levels of diversifying selection, and were not selected differently than predicted antigenic epitopes in *B*. *malayi* and *O*. *volvulus*. Measurements of ALT-2 evolvability made by χ^2^ analysis between alleles that were stable (*O*. *volvulus* and *B*. *malayi*) and those that were under diversifying selection (*W*. *bancrofti* and *L*. *loa*) indicated significant (*P*<0.01) deviations from a normal distribution for both *W*. *bancrofti* and *L*. *loa*. The relationship between evolvability and selection in *L*. *loa* followed a second order polynomial distribution (R^2^ = 0.89), indicating that the two factors relate to one another in accordance with an additional unknown factor. Taken together, these findings indicate discrete evolutionary drivers acting on ALT-2 of the four organisms examined, and the described variation has implications for design of novel vaccines and diagnostic reagents. Additionally, this represents the first mathematical description of evolvability in a naturally occurring setting.

## Introduction

Filarial parasites are causative agents of the neglected tropical diseases river blindness (RB; *Onchocerca volvulus*), African eye worm disease (EWD; *Loa loa*), and lymphatic filariasis (LF; *Wuchereria bancrofti*, *Brugia malayi*). While their mortality rates are low, these diseases carry significant burdens as measured by disability-adjusted life years (DALYs) [1, 2]. Long-term sequellae including loss of mobility and eyesight stem at least in part from immunopathologic processes, and therefore persist even after successful eradication of adult worms from infected patients [2, 3]. Vaccination is thought to be the most promising approach to elimination of RB, EWD, and LF, but development is complex due to the potential for immunopathology. While strides have been made in reducing disease prevalence by employing preventative chemotherapy and transmission control strategies, these approaches are not ideal long-term solutions. Reaching drug administration levels sufficient to disrupt transmission can be logistically challenging because of periodic population fluctuation leading to inaccurate census data, noncompliance, the human resources necessary to manage and monitor programs, and the possibility of selecting for benzimidazole and ivermectin resistance [4–8]. Given the challenges inherent to mass drug administration programs, an important goal to prevent and control these infections has been to develop effective anti-filarial vaccines.

The abundant larval transcript protein ALT-2 has been described as a potential candidate for a subunit vaccine for *B*. *malayi*, and its homologues in *O*. *volvulus* (SLAP1), *W*. *bancrofti* (ALT-2), and *L*. *loa* (ALT-2 or ‘larval allergen’) hold the same potential [9, 10]. The appeal of ALT-2 as a protective antigen includes its immunogenicity as demonstrated by seropositivity of LF patients and “endemic normals” [11–14], its lack of predicted cross-reactivity with human epitopes [11], and its expression only in the larval stages to avoid the immunopathology associated with adult worms [15]. Vaccination-(homologous) challenge studies carried out with *B*. *malayi* in gerbils, mice, and rhesus macaques demonstrated that purified ALT proteins mediated complete protection or substantial reductions in parasite load [11, 15–16].

Despite this promise, ALT proteins have a characteristic variable acidic domain (VAD), which introduces a potential complication for vaccine and diagnostic testing applied to heterologous strains. We thus sought to characterize the type of natural selection acting on ALT-2 of *O*. *volvulus*, *L*. *loa*, *W*. *bancrofti* and *B*. *malayi* in order to predict whether ALT-2 is a suitable protective or diagnostic antigen across filarial parasites. Detection of diversifying selection indicates evolutionary adaptation by increasing sequence variation, and purifying selection indicates adaptation by favoring sequence conservation. Additionally, we devised a calculation for the capacity for ALT-2 to change in response to evolutionary pressures, or its evolvability [17]. The type of evolutionary pressure and its location within a given protein has major implications for the ability of a pathogen to evade immune responses or diagnostic detection, and thus should be considered during vaccine development.

## Materials and Methods

### Nucleotide Sequences

All nucleotide sequences for ALT-2 homologues were mined from GenBank using the following accessions: AAC35355, ADC54122, EJW81953, EJW75260, EJW69923, EJW71405, AAG31481, EJW77960, EJW71128, ACB70201, AAG16997, AAB69626, AAD27588, AAC79423, AAC79126, AAA84910, AAD27587, EFO14886, XP_003149183, EFO18446, EFO13555, XP_003150514, EFO15914, XP_003151340, EFO12729, XP_003148155, AAN62757, EDP34936, XP_001896203, EDP29031, AAN85136, XP_001902123, EDP34327, XP_001896826, AAF01225, AAF01224, EDP29032, XP_001902122, AAB41884 [10, 18–21]. Clinical and geographic information for the source material of sequences was often, but not always, unreported. Established source information includes geographic origins for parasites including Cameroon, Central African Republic, Ecuador, India, Mali, Papua New Guinea, and Uganda. Isolates originated from a range of biological origins including microfilaremic patients, symptomatic patients, insect vectors, and experimentally infected animals.

### Selection Analysis

Bayesian models of sequence evolution in the Selecton v2.4 software suite [22] were used to detect diversifying, neutral, or purifying selection acting on ALT-2 of *B*. *malayi*, *W*. *bancrofti*, *L*. *loa*, and *O*. *volvulus*. Aligned sequences from each species were individually interrogated using the M8 model and the mechanistic-empirical combined (MEC) model [23, 24]. The mechanistic model M8 uses maximum likelihood methods where scoring is weighted by different probabilities for transitions and transversions, codon bias, and among-site rate variation to estimate the proportion of codons with ω values of <1 (the beta distribution *p*_0_) and the proportion with ω values of ≥1 (ω*s*). Sites with ω values of <1 reflect stabilizing selection, and sites with ω values of ≥1 reflect either neutral or diversifying selection. Position-specific as well as global inferences about the forms of selection acting on the protein can thus be made. The MEC model additionally accounts for differing biochemical impact of amino acid substitutions by assigning a higher site-specific *K*_*a*_ value at a position with physicochemically radical amino acid changes in polarity or charge than at a position with less extreme mutations. The MEC model is thus more likely to indicate diversifying selection based on a smaller proportion of sites with ω values substantially greater than 1 (with or without a high global ω value) compared to the more conservative M8 model.

### Statistical Analysis

The statistical significance of global selection on each protein was determined by likelihood ratio test between the M8 model and a null model (M8a) (Selecton v2.4). The M8a model simulates neutral or stabilizing selection based on negative deviations from a fixed ω*s* value of 1. In the absence of diversifying selection, the difference between the likelihood scores generated by the M8 and M8a models follows a normal χ^2^ distribution [25]. Significant deviations are indicative of global diversifying selection.

### Protein Informatics Analysis

Nucleotide sequences were translated using ExPasy Translate [26]. Signal peptides were identified by SignalP [27]. Secondary and tertiary structural predictions for topological projection were made using Swiss-Model [28], Phyre2 [29], m4T [30], RaptorX [31], ITASSER [32], HHpred [33], and ModWeb [34] using the Protein Model Portal and Swiss-Model Workspace. Antigenicity predictions for the ALT-2 consensus sequences (S1 Dataset) were made using Kolaskar and Tongaonkar residue antigenicity scales as implemented by EMBOSS Antigenic [35] and the antigenic tool at http://imed.med.ucm.es/Tools/antigenic.html.

### Phylogenetic Analysis

The phylogenetic relationship of the ALT-2 consensus sequences was inferred using the neighbor-joining and maximum parsimony methods [36, 37]. Consensus trees representing 500 bootstrap replicates were generated [38**]**, and the evolutionary distances were computed using the Poisson correction method [39]. Phylogenetic analyses were conducted in MEGA 4.02 [40].

### Evolvability Analysis

Pairwise alignments (see Table 1) using the consensus amino acid sequences for ALT proteins of each species were generated using Clustal Omega [41]. The ω values representing selection for each amino acid residue in the ALT proteins (Selecton v2.4) were recorded and applied to each pairwise alignment. Gaps between aligned segments were assigned an ω value of 1.0 for each missing residue to allow for evolvability calculations as compared to the ω value for the gapped residues. For traits encoded by homologous genes that are not evolvable, selection at each individual site can be expected to follow a normal distribution in different species. Genetic backdrops (*i*.*e*., distinct species) that facilitate differential selection to occur on a given trait would be expected to deviate from a normal distribution at each site. The latter can be measured when homologous traits are under purifying selection in some species (treated as an expected ω value) and diversifying selection in others (treated as an observed ω value). Significant deviation from the normal distribution is indicative of evolvability. In order to calculate evolvability at each site, pairwise χ^2^ goodness-of-fit analyses were conducted on ω value distributions for ALT proteins of *O*. *volvulus*, *L*. *loa*, *W*. *bancrofti*, and *B*. *malayi* as indicated in Table 2 (Origin v. 9.0). The negative control analysis performed between *B*. *malayi* and *O*. *volvulus* represents a series of null model calculations at each amino acid site that are expected to represent a normal distribution (*i*.*e*., no significant evolvability), and the positive control analysis between *L*. *loa* and *W*. *bancrofti* represents a series of calculations at each amino acid site that are expected to deviate from normal distribution (*i*.*e*., significant evolvability). The mathematical relationship between selection and evolvability was determined by scatter plot analysis.

**Table 1:** Pairwise Evolvability (E) Analysis Design.

Analysis	Species 1 (expected)	Species 2 (observed)	Experimental Role
1	*B*. *malayi*	*W*. *bancrofti*	*W*. *bancrofti* E Analysis
2	*O*. *volvulus*	*W*. *bancrofti*	*W*. *bancrofti* E Analysis
3	*B*. *malayi*	*L*. *loa*	*L*. *loa* E Analysis
4	*O*. *volvulus*	*L*. *loa*	*L*. *loa* E Analysis
5	*L*. *loa*	*W*. *bancrofti*	Control (+)^a^
6	*O*. *volvulus*	*B*. *malayi*	Control (-)^a^

^a^The negative control analysis represents null model calculations at each amino acid site that are expected to represent a normal distribution (*i*.*e*., no significant evolvability), and the positive control analysis represents calculations at each amino acid site that are expected to deviate from normal distribution (*i*.*e*., significant evolvability).

**Table 2:** *In vivo* Immunogenicity and Evolvability.

Immunogenic Epitope	AE1 Region 1–21	AE2 Region 21–50	AE3 Region 104–128	Peptide 4 Region 55–68	Peptide 6 Region 73–91
Epitope Sequence^a^	MNKLLIAFGLVILLVTLPCAS	SESDEEFDDGSNDETDDKEDEGNSEGGDEY	CVIERKNNGKLEYSYCAPEAGWQCA	EVVETDGKKKECSS	YDQREPQAWCRPNENQSWT
Consensus Sequence^b^	MNKLLIVFGLIILFATPLYAK	KQSNEEEEEMSNEEEKENGSKEEEDEEDYS	EYTAKGEFVKTDGKKKQCDSHVACY	EDEEKNESGEKEDE	RSKEEEEDEDEDGGEEDED
Evolvable residues (*P*<0.05)	2/21	0/30	1/24	0/13	0/18
Organism	*W*. *bancrofti*	*W*. *bancrofti*	*W*. *bancrofti*	*B*. *malayi*	*B*. *malayi*
Reference	[44]	[44]	[44]	[11]	[11]

^a^The listed sequences are those reported in the appropriate reference.

^b^Consensus sequence reflects the most frequent residues across all strains of the noted species at each site, determined in our current analysis.

## Results

### Selection Analysis

**Fig 1:**
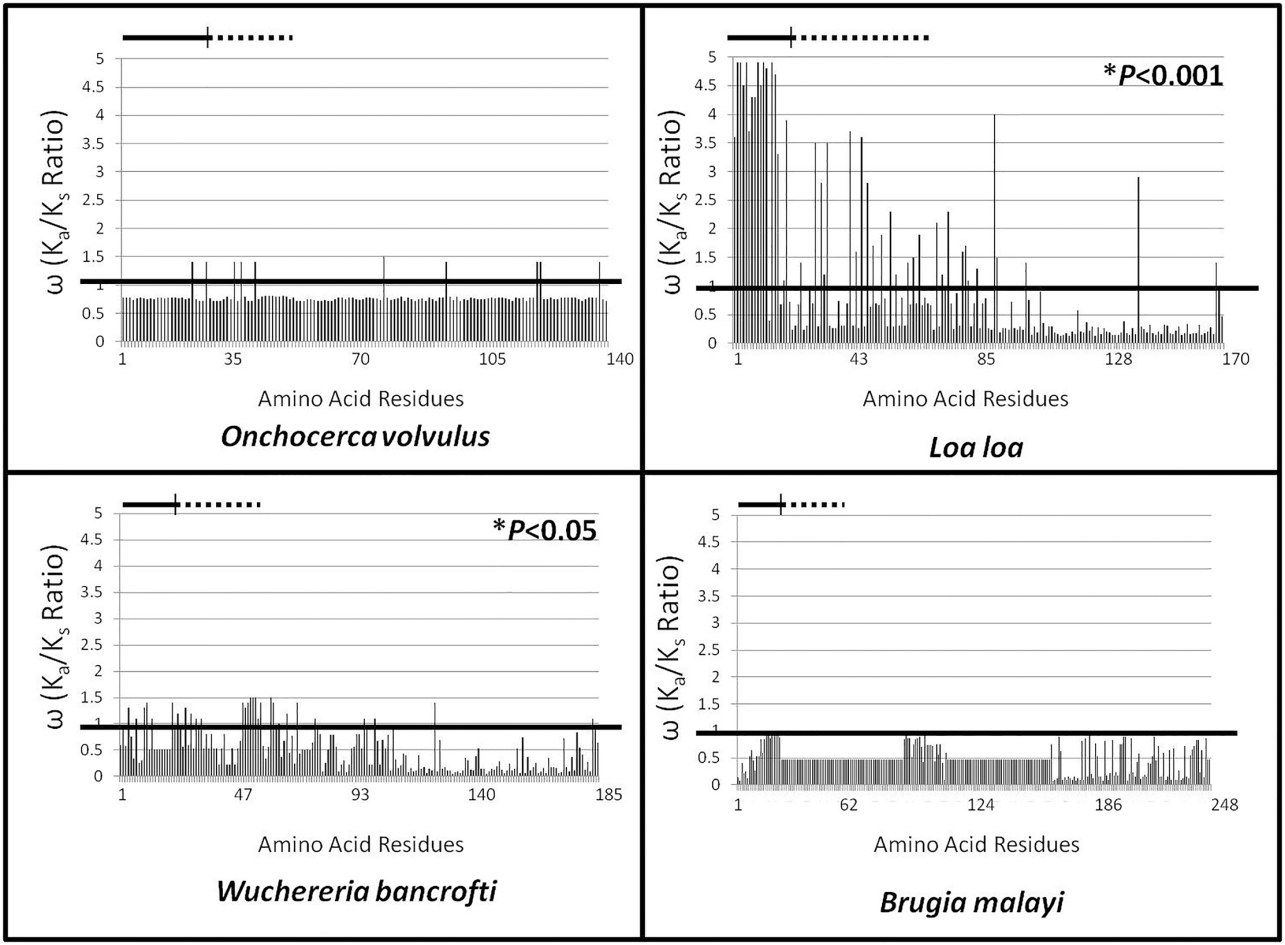
Evolutionary Selection Analysis. Calculated ω values are presented for each amino acid residue (X axis, marked by position number) for ALT-2 from the indicated species using the M8 model. The cutoff ω value of 1 is depicted as a black line; values extended above the line represent residues under diversifying selection, and values below the line represent residues under purifying selection. Statistically significant diversifying selection was detected globally in ALT-2 of *L*. *loa* (*P*<0.001) and *W*. *bancrofti* (*P*<0.05). Residues encompassing the signal peptide are beneath the solid lines, and those encompassing the VAD are beneath the dashed lines.

Statistically significant diversifying selection was detected globally in ALT-2 of *L*. *loa* (*P*<0.001) and *W*. *bancrofti* (*P*<0.05) using the M8 model (Fig 1A). Diversity was notably highest for the predicted signal peptide of *L*. *loa*, but not *W*. *bancrofti*. Diversifying selection was found acting on a small number of individual residues in *O*. *volvulus*; however, globally the effect was not significant. No individual residues in ALT-2 of *B*. *malayi* were found to be under diversifying selection. No patterns of diversity or conservation relating to geographic or clinical origin were apparent.

### Protein Informatics Analysis

**Fig 2:**
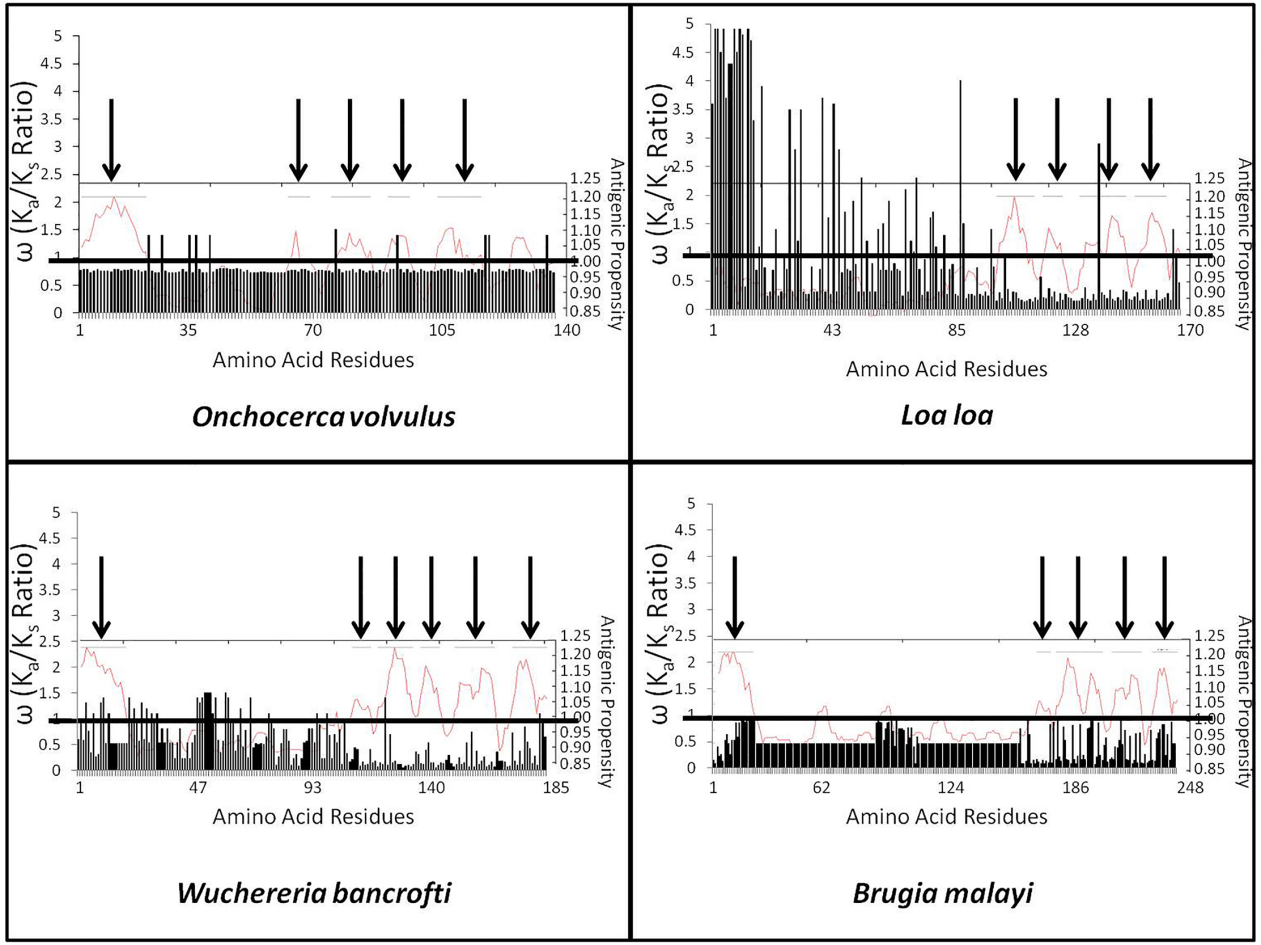
Antigenicity and Selection Projection. Calculated ω values using the M8 model (Y axis, bar graph) are projected onto graphical measurements of antigenicity predictions (Z axis, line graph) across all sites in ALT-2 (X axis, marked by position number). Predicted antigenic epitopes are denoted with arrows. The cutoff ω value of 1 is depicted as a black line; values extended above the line represent residues under diversifying selection, and values below the line represent residues under purifying selection. Areas under diversifying selection in ALT-2 of *L*. *loa* and *W*. *bancrofti* have minimal overlap with predicted antigenicity.

ALT-2 proteins from all four species were predicted to contain signal peptides. Structural predictions could not be made on the consensus sequences with any known algorithms due to the proportion (>50%) of disordered residues and the below threshold (0.0001) alignment with currently solved structures. Topological projections of heavily selected residues were therefore not possible. Each consensus sequence features antigenic epitopes, ranging from 4 (*L*. *loa*) to 6 (*W*. *bancrofti*). Antigenic epitopes did not coincide with regions of diversifying selection (Fig 2).

### Phylogenetic Analysis

**Fig 3:**
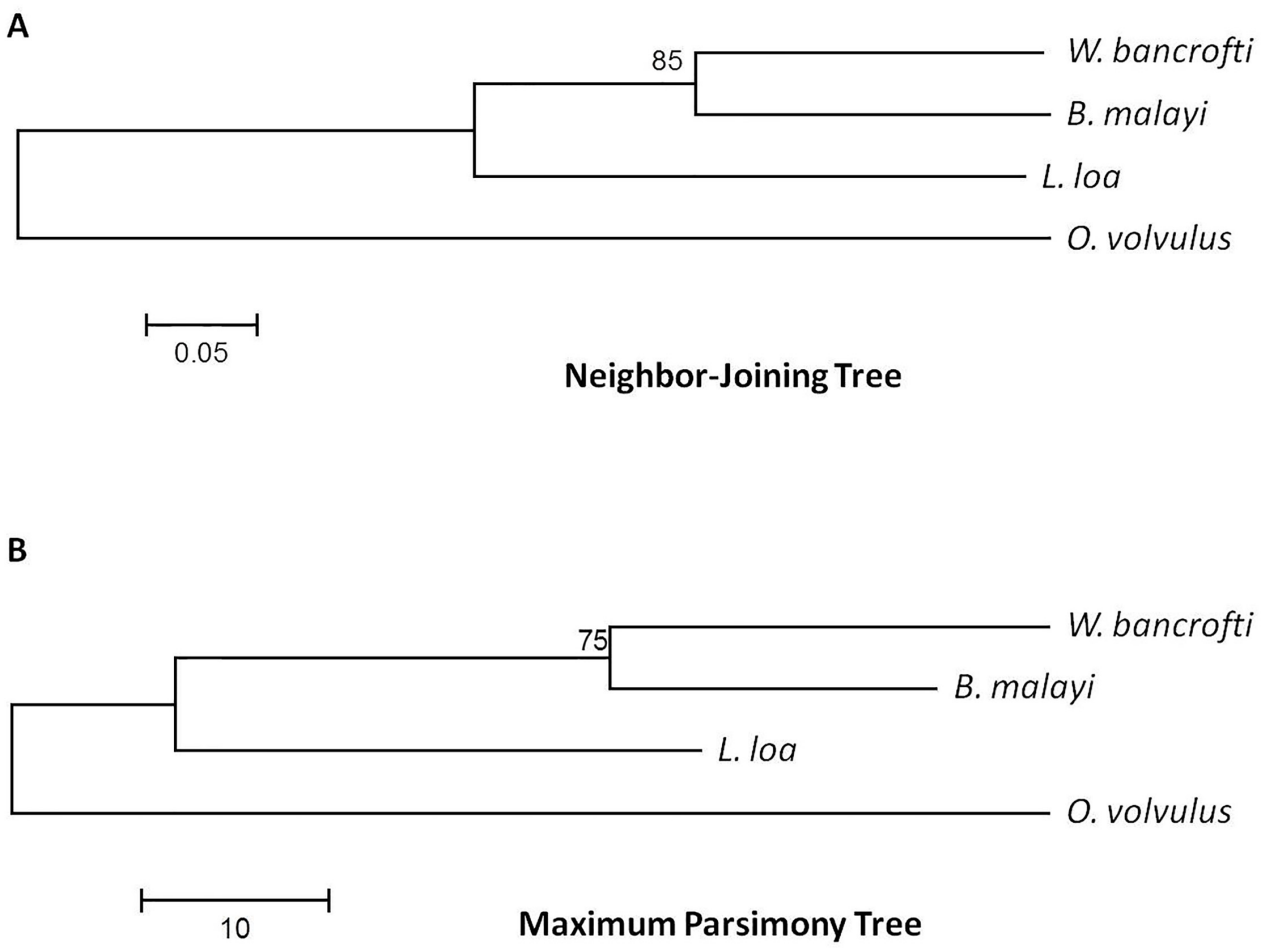
Phylogenetic Analysis. The evolutionary history of ALT-2 consensus sequences from *B*. *malayi*, *W*. *bancrofti*, *O*. *volvulus*, and *L*. *loa* was inferred using (**A**) the Neighbor-Joining method and (**B**) the Maximum Parsimony method (N = 500 bootstrap replicates each). Regardless of method, the branching order was the same and was consistent with ribosomal RNA-derived branching order.

Phylogenetic analysis based on ALT-2 consensus sequences using either neighbor-joining or maximum parsimony methods revealed similar relationships between species as trees based on ribosomal RNA [42, 43] (Fig 3).

### Evolvability Analysis

**Fig 4:**
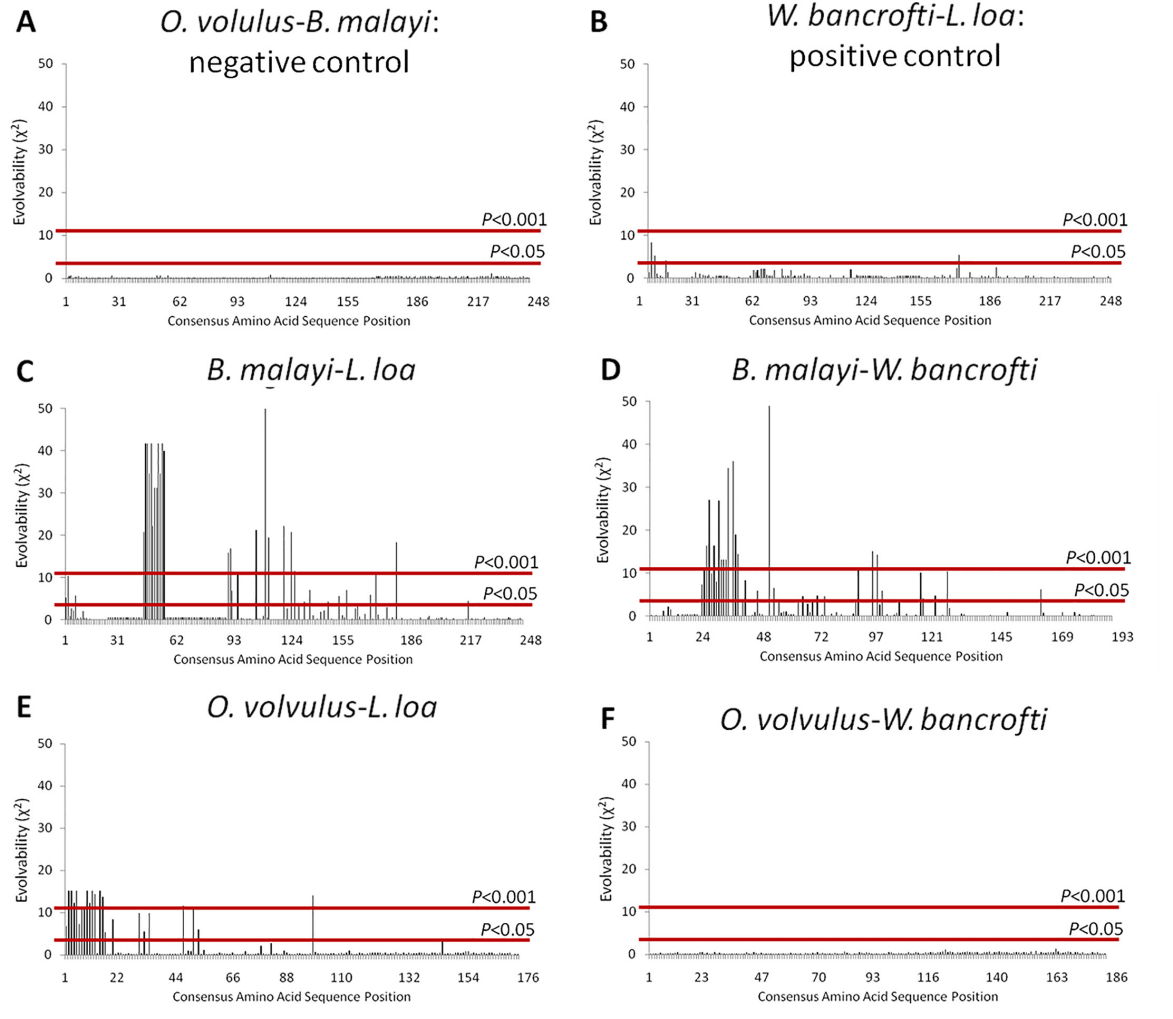
ALT-2 Evolvability in Filarial Parasites. Calculated evolvability (E) values (Y axis) are presented for each amino acid residue (X axis) for ALT-2 from the indicated pairs of species using the calculated ω values from each organism at each site. Cutoffs for significant deviations between conserved (normal distribution) and diversified sequences are shown as red lines (*P*<0.05 or *P*<0.001); values extended above the line represent residues that are significantly evolvable. Goodness-of-fit analyses between the two conserved ALT-2 sequences (*O*. *volvulus* and *B malayi*, **A**) and the two diversified ALT-2 sequences (*L*. *loa* and *W*. *bancrofti*, **B**) served as positive and negative controls, respectively. Multiple residues of *L*. *loa* were significantly evolvable when examined for deviation from *B*. *malayi* (**C**) or *O*. *volvulus* (**E**). Multiple residues of *W*. *bancrofti* were significantly evolvable when examined for deviation from *B*. *malayi* (**D**), but not *O*. *volvulus* (**F**).

**Fig 5:**
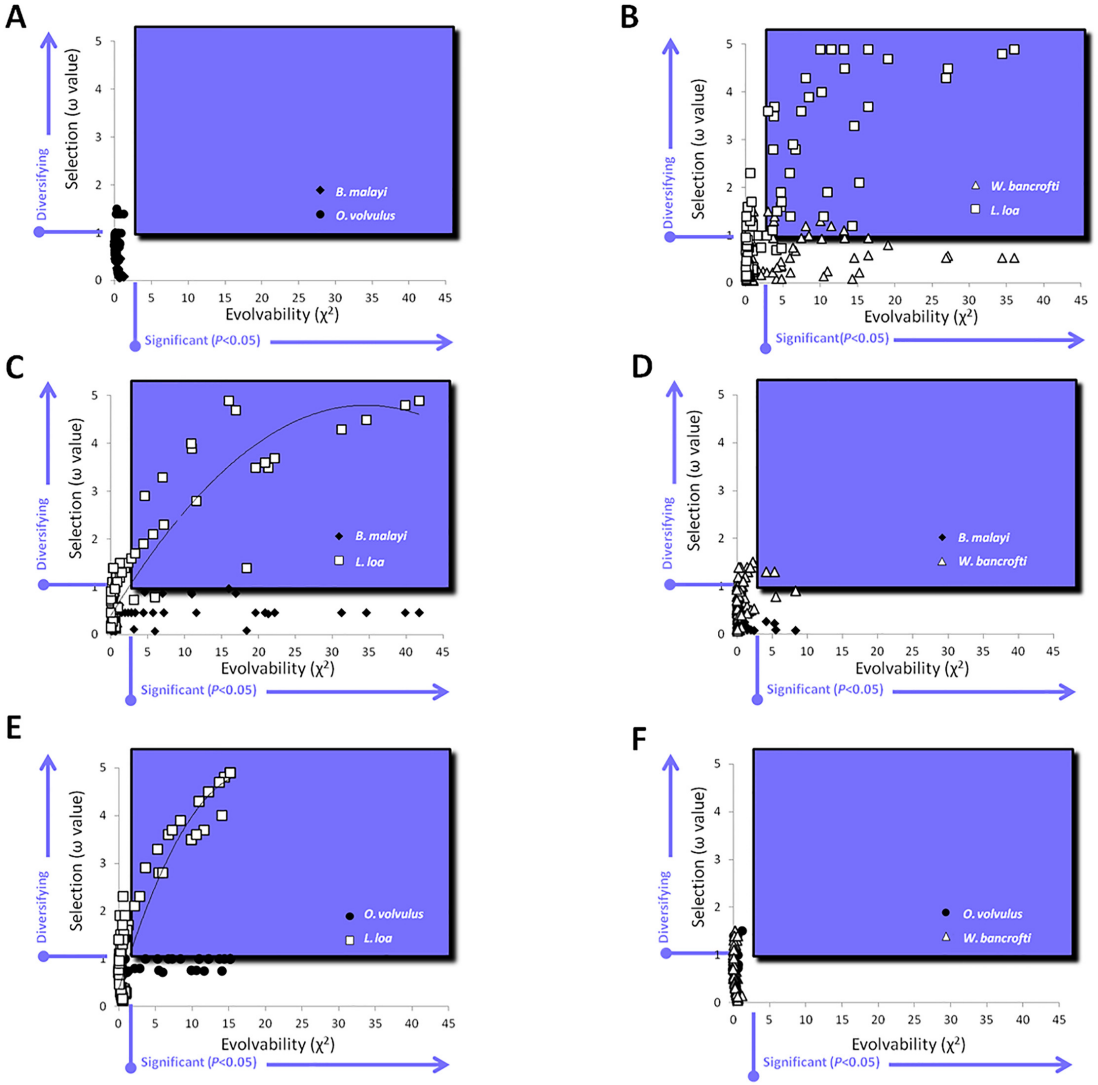
Selection and Evolvability of ALT-2. Values for selection (ω) and evolvability (E) were calculated for each amino acid residue in the ALT-2 sequence. The single E value was plotted against the ω value for pairwise comparisons between all species. Residues that were both significantly evolvable and under diversifying selection appear in the blue-shaded boxes. The two conserved ALT-2 sequences (*O*. *volvulus*, [filled circles] and *B malayi* [filled diamonds], **l A**) and the two diversified ALT-2 sequences (*L*. *loa* [open squares] and *W*. *bancrofti* [open triangles], **B**) served as positive and negative controls, respectively. Selection and evolvability of ALT-2 from *L*. *loa* followed a second-order polynomial distribution when compared to either *B*. *malayi* (**C**) or *O*. *volvulus* (**E**). Only a small number of residues in *W*. *bancrofti* were both diversified and evolvable when compared to *B*. *malayi* (**D**), but none were detected when compared to *O*. *volvulus* (**F**).

Positive (*L*. *loa* vs. *W*. *bancrofti*) and negative (*O*. *volvulus* vs. *B*. *malayi*) controls displayed expected χ^2^ distributions; namely, distributions did not differ significantly between negative controls while positive controls did (*P*<0.01). Distributions between species where ALT proteins are negatively selected (*O*. *volvulus* vs. *B*. *malayi*, “expected”) and those where ALT proteins are under diversifying selection (*L*. *loa* vs. *W*. *bancrofti*; “observed”) showed that the selection values for ALT proteins of *L*. *loa* deviated from the normal distributions generated by either *B*. *malayi* or *O*. *volvulus*. Significant deviation from the normal distribution is indicative of evolvability. Numerous residues were found to be significantly (*P*<0.001) evolvable in *L*. *loa* when calculated relative to either *O*. *volvulus* or *B*. *malayi*, whereas only a small number of residues from *W*. *bancrofti* were found to be significantly (*P*<0.05) evolvable, and only when calculated relative to *B*. *malayi*. (Fig 4). The mathematical relationship between selection and evolvability followed a second order polynomial distribution for *L*. *loa* (Fig 5C and 5E), but not for *W*. *bancrofti* (Fig 5D).

## Discussion

The abundant larval transcript proteins are conserved in all filariae, but their function remains unknown. Expression during infection of human hosts clearly occurs given that seropositive patients frequently have reactive antibodies to ALT-2 [44]. Previous studies have shown that purified ALT-2 of *B*. *malayi* elicits a protective immune response in mice and jirds, indicating that these proteins may potentially serve as the basis for subunit vaccines [45]. Evolutionary selection analysis of the ALT-2 proteins from four prominent human pathogens of the *Filarioidea* was performed to better evaluate their long-term potential as protective antigens. Findings of globally acting natural selection favoring sequence diversity in *L*. *loa* and *W*. *bancrofti*, but not *O*. *volvulus* or *B*. *malayi*, were unexpected. These data indicate that not only are ALT-2 proteins selected differently by distinct filarial species, but that selection does not correlate with target body site (eye versus lymphatic system) or clinical presentation (RB/EWD versus LF). While inferring selection based on ω ratios allows consideration only of changes in protein sequence rather than changes in gene expression level or timing, this limitation of the analysis would result in false-negative findings rather than false-positive [24]. In contrast, reported elevated rates of diversity among close lineages (such as between strains) being falsely attributed to selection would be expected to generate a uniform pattern across homologous genes [46]. Given that we observed positive selection in *L*. *loa* and *W*. *bancrofti*, it is unlikely that diversity is generated by changes in gene expression. Additionally, findings of purifying selection acting on ALT-2 of *B*. *malayi* and *O*. *volvulus* suggests that the rate of drift affecting the encoding gene is not substantial enough to generate false detection of positive selection. No patterns of diversity or conservation relating to geographic or clinical origin were apparent; however, additional analysis with greater repeat measures for each variable are required to fully evaluate this conclusion.

The consensus sequences of ALT-2 proteins from all four species were used to draw phylogenetic trees using both neighbor-joining and maximum parsimony methods (500 bootstrap replicates each). Branching patterns were consistent with relationships described for trees generated with ribosomal RNA sequence. This is largely suggestive of sequence features that are inherited by descent, and the diversity observed in *L*. *loa* and *W*. *bancrofti* does not appear to drive them toward a convergent point. In contrast, significant evolvability was observed at a small number of sites for *W*. *bancrofti* and many sites for *L*. *loa*, which are more closely related by 18S rRNA sequence than to *O*. *volvulus*. If the capacity to measurably evolve in response to diversifying selection is a heritable trait [17, 47], it has either been lost in *B*. *malayi* or does not have an appropriate evolutionary driver compelling it to manifest in this species.

Reports of evolvability for macroscopic, free living organisms have included numerous accepted definitions; as a consequence, parameters of measurable evolvability can be diverse [17]. Parasitic or endosymbiotic organisms present ideal study systems to examine evolvability due to their comparably streamlined genomes and the selective pressure of survivorship as opposed to competition. The measureable differences in selection acting on the products of *alt2* homologues of closely-related *Filarioidea* species suggested that the evolvability of ALT-2 is context-dependent rather than intrinsic. We thus sought to quantify evolvability as deviation from a normal distribution derived from the K_a_/K_s_ ratios of stable alleles under purifying or neutral selection (*i*.*e*., ALT-2 homologues of *B*. *malayi* and *O*. *volvulus*) at each amino acid site. For each amino acid site, significant (χ^2^; *P*<0.05) deviations derived from the K_a_/K_s_ ratios of variable alleles under diversifying selection (*i*.*e*., ALT-2 homologues of *W*. *bancrofti* and *L*. *loa*) demonstrate evolvability at that site. To our knowledge, the use of stable alleles in one organism as the reference control for variant alleles of the homologue in another organism is a novel way of measuring naturally occurring evolvability of a trait in real time. The mathematical relationship between selection and evolvability for ALT-2 of *L*. *loa* was a univariant second-order polynomial when calculated against either *B*. *malayi* or *O*. *volvulus* (Fig 5). No such relationship was found between selection and evolvability for ALT-2 of *W*. *bancrofti*, or in the positive control evolvability analysis comparing ALT-2 selection values from *W*. *bancrofti* and *L*. *loa* against each other. The second-order polynomial distribution observed for *L*. *loa* indicates that evolvability values are a function of selection values. Taken together, this indicates that the significant evolvability of ALT-2 for *L*. *loa* which manifests as genetic diversity is generated by the influence of diversifying selection, and this is not the case for *W*. *bancrofti*.

Antigenicity analysis made on the consensus sequences revealed that a majority of the sites predicted to comprise immunogenic epitopes clustered on the carboxy-terminal half of ALT-2 for all species. The antigenicity prediction algorithms are largely based on hydrophilicity and polarity values across short stretches of sequence that are not predicted to be in low-complexity regions. Consideration of tertiary structure is ideally used to ensure that a predicted epitope is not buried and inaccessible to antibodies; however; this level of analysis was not possible with ALT-2 sequences because the tertiary structure could not be predicted. The *in vivo* relevance of antigenicity prediction for ALT-2 proteins was established by Madhumathi *et al*. by exposing spleen-derived T cells from *B*. *malayi*-immunized mice to peptides representing predicted antigenic epitopes and achieving high levels of activation and proliferation [11]. This is critical to the interpretation of our analysis because the most immunogenic epitopes were strikingly devoid of diversifying selection, and a majority had at most a single residue with an ω value greater than 1 (Fig 3). Peptides observed to stimulate T cell activation in hyperimmune animals [11] were under purifying selection in our analysis. Further, sequences with demonstrated T and B cell antigenicity [11, 44] had only 2 of a collective 106 residues that were significantly evolvable (Table 2). This suggests that the driver of diversifying selection and evolvability in *L*. *loa* and *W*. *bancrofti* is unlikely to be host immunity, as has been observed for many pathogens [47, 48]. Alternatively, if the ability to rapidly alter protein sequence is functionally beneficial, diversifying selection will occur. The remarkably high ω values and evolvability measurements in the signal peptide of *L*. *loa* may give an indication that altering the rate or destination of ALT-2 secretion is advantageous to this organism, but the same ability does not add any further benefit to *O*. *volvulus* or *B*. *malayi*. While this region is under diversifying selection in *W*. *bancrofti*, it is not substantially different from other regions of the protein. Interestingly, *L*. *loa* was the only one of the four species not to have an antigenic epitope predicted within the signal peptide. This is striking, given the close association with this site for protective immunity against *B*. *malayi* in experimental challenge studies [49]. It is also notable that the VAD, contrary to prediction, was not positively selected in *O*. *volvulus* or *B*. *malayi*, and had ω values similar to or lower than the signal peptide in *W*. *bancrofti* and *L*. *loa* (respectively). It is further noteworthy that the VAR did not correspond with any predicted antigenic epitopes, similarly suggesting that the observed variation is functionally favorable and not necessarily related to immune escape.

These findings are consistent with reports of protective immunity following immunization of jirds with ALT-2 from *W*. *bancrofti* and *B*. *malayi*, and with higher levels of seropositivity against *W*. *bancrofti* ALT-2 in endemic normal patients relative to those who are microfilaremic or experiencing chronic pathology [10, 45, 50]. It is potentially consistent with reports of immunity to a 23 kDa antigen corresponding with microfilaremia relative to overt pathology in loiasis patients [51]. Collectively these findings illustrate that evolutionary selection acting on ALT-2 proteins is not uniform across members of the *Filarioidea*, and that it may be driven by protein function rather than host immunity. Relevant protein activities are unlikely to involve a simple association between ALT-2 and tissue tropism for either the eye or the lymphatic system, or development of clinical disease. The function of ALT-2 remains to be characterized. Perhaps unexpectedly, these findings indicate that the observed diversity does not exclude ALT-2 as a promising protective antigen, consistent with human serological and animal vaccination-challenge studies. Our analysis indicates that the driver of diversity is not likely to be immune selection; therefore, vaccine escape can be predicted as relatively minor. Taken together, these conclusions support the development of this protein as a candidate subunit vaccine.

## Supporting Information

S1 Dataset S1 Dataset contains the consensus sequences for ALT-2 from each nematode species examined in FASTA format. (FASTA)
